# More rapid climate change promotes evolutionary rescue through selection for increased dispersal distance

**DOI:** 10.1111/eva.12004

**Published:** 2012-09-25

**Authors:** Jeroen Boeye, Justin M J Travis, Robby Stoks, Dries Bonte

**Affiliations:** 1 Terrestrial Ecology Unit, Ghent UniversityGhent, Belgium; 2 Institute of Biological and Environmental Sciences, University of AberdeenAberdeen, UK; 3 Laboratory of Aquatic Ecology and Evolutionary Biology, KU Leuven LeuvenBelgium

**Keywords:** climate change, evolution of dispersal kernels, evolutionary rescue, individual-based model, plants, range expansions

## Abstract

Species can either adapt to new conditions induced by climate change or shift their range in an attempt to track optimal environmental conditions. During current range shifts, species are simultaneously confronted with a second major anthropogenic disturbance, landscape fragmentation. Using individual-based models with a shifting climate window, we examine the effect of different rates of climate change on the evolution of dispersal distances through changes in the genetically determined dispersal kernel. Our results demonstrate that the rate of climate change is positively correlated to the evolved dispersal distances although too fast climate change causes the population to crash. When faced with realistic rates of climate change, greater dispersal distances evolve than those required for the population to keep track of the climate, thereby maximizing population size. Importantly, the greater dispersal distances that evolve when climate change is more rapid, induce evolutionary rescue by facilitating the population in crossing large gaps in the landscape. This could ensure population persistence in case of range shifting in fragmented landscapes. Furthermore, we highlight problems in using invasion speed as a proxy for potential range shifting abilities under climate change.

## Introduction

The impact of global change on species varies over a range of factors. There is a consensus that global temperatures have been drastically increasing over the last decennia and that this trend will not be halted in the forthcoming decades (IPCC 2007). How fast this global warming will take place is difficult to predict because of uncertainties in upcoming human impact, which may either speed-up or slow-down the process (Pereira et al. 2010). In addition, there is evidence that certain regions on this planet are more sensitive to climate change than others (Thomas et al. 2004; Loarie et al. 2009). Similarly, the rate of climate change will be perceived differently by different species because of interspecific differences in thermal sensitivity, dispersal and generation time (Berg et al. 2010) generating a wide variety in responses (Chen et al. 2011). The current rate of climate change, in combination with other global environmental impacts forces organisms to either adapt, migrate or go extinct (Visser 2008). While there is ample evidence that species from a wide range of taxonomic groups are moving polewards and to higher elevations (Parmesan 2006; Thomas 2010; Chen et al. 2011), a large proportion of species are still expected to become extinct (Thomas et al. 2004; Pereira et al. 2010). The combined action of habitat fragmentation and climate change rates has indeed been demonstrated to be a deadly cocktail for the persistence of species (Travis 2003; Warren et al. 2011).

A wide range of models have been developed to predict future species ranges to understand the biological effect of, and responses to, climate change. Correlative approaches that determine climate envelopes are widely used (Hampe 2004), but there are several limitations in the approach, amongst others neglecting dispersal as a fundamental process in range shifting. Analytical models like reaction-diffusion-(Shigesada and Kawasaki 1997), integro-difference-(Neubert and Caswell 2000) or (semi-)mechanistic models (Katul et al. 2005) all do incorporate the dispersal process in one way or another but typically only consider populations in spatiotemporally stable environments. While there have been some attempts to parameterize simple analytical models to infer range expansion (Bullock et al. 2008), there has recently been an increased appreciation of individual-based models to generate more generic insights into the mechanisms by which global change might impact the capacity of a population to spread and persist (Brooker et al. 2007; Phillips et al. 2008; Mustin et al. 2009; Kubisch et al. 2010; Fronhofer et al. 2011). These models account for the presence of spatially shifting climate windows and, in some cases, focus solely on ecological dynamics (Brooker et al. 2007; Mustin et al. 2009) while in others, eco-evolutionary responses are explored (Phillips et al. 2008; Kubisch et al. 2010; Fronhofer et al. 2011). However, none of these studies have looked into the impact of the rate of climate change when dispersal is allowed to evolve. Dispersal has been repeatedly shown to evolve under the influence of landscape changes (e.g., Bonte and Lens 2007; Cheptou et al. 2008; Hanski and Mononen 2011), and such evolutionary changes may induce evolutionary rescue (Ronce 2007). Moreover, the use of dispersal distance in a spatially explicit context rather than dispersal propensity in combination with different rates of climate change is expected to yield novel and more realistic insights of eco-evolutionary mechanisms related to range shifting under climate change.

The significance of evolution as an important driving process of range expansions is currently recognized by both empirical (e.g. Thomas et al. 2001; Phillips et al. 2006, 2008; Léotard et al. 2009) and theoretical work (e.g. Garcia-Ramos and Rodriguez 2002; Travis and Dytham 2002). The evolution of dispersal rate has received considerable interest and generated insights on range shifts and range border formation. In theoretical work by Dytham (2009), dispersal rates have for instance been shown to increase towards range margins with increased environmental and demographic stochasticity, but to decrease if habitat gradually becomes less available. Results from a simulation model developed by Phillips (2012) suggest that recent range shifts could even promote the formation of stable range edges because more dispersive individuals experience environmental gradients more intensively. However, a different model suggests that when dispersal costs at range margins become too high, selection against dispersal may eventually induce range contraction (Kubisch et al. 2010).

Most studies do not consider the evolution of dispersal distance, although high dispersal rates are known to evolve at range borders and to induce evolutionary rescue in theoretical studies (Travis et al. 2009; Bonte et al. 2010; Fronhofer et al. 2011). While we do not doubt that models inferring dispersal rate by implementing either nearest neighbour or global dispersal provide fundamental insights into dispersal evolution, we emphasize that in reality dispersal kernels as well as emigration rate will be under selection, which will exert pressure especially on those traits determining dispersal distance (e.g. Bonte et al. 2009; Barton et al. 2011). For instance, in plants, all seeds disperse to some degree, but selection on traits like seed weight, plant height or specific dispersal structures (from fruits to wings; see Bonte et al. 2012) will eventually determine how long seeds can remain airborne, and as such how far they can be potentially spread (Cousens et al. 2008). Given the importance of dispersal distance in range expansion (Simmons and Thomas 2004; Phillips et al. 2008) or spatial populations dynamics (Leibold et al. 2004; Cousens et al. 2008), it is surprising that the evolution of dispersal kernels has only received marginal attention (Ronce 2007).

Evolution at range borders results from two complementary processes, that is, natural selection within populations and the spatial sorting of genotypes near expanding range margins (Shine et al. 2011). Spatial sorting increases the frequency of dispersive genotypes near the expanding range edges based on the standing variation in populations rather than by mutations in the edge populations. This is because dispersive genotypes tend to be overrepresented near the expanding front and are thus more likely to mate with each other (the Olympic village effect) (Phillips et al. 2008). The magnitude of both natural selection and spatial sorting will be influenced by the rate of climate change because variation here-in will determine the availability of unoccupied but suitable habitat beyond the current range border and mortality of low-dispersive individuals near the trailing edge of the range (Phillips et al. 2008; Dytham 2009). Regardless of the exact rate of climate change, we expect the population density to increase from the expanding front onwards. Dytham (2009) showed that such gradients in population dynamic parameters can influence local selection pressures and result in a gradient in dispersiveness.

Given the expected variation in how different species perceive the rate of climate change, it is reasonable to assume that different species will show different ecological, but also evolutionary responses towards climate change speed. A fast climate change is expected to be worse than a slow one because it reduces the time available for species to adapt to the new environment or to shift their range to cooler regions (Visser 2008). By developing a generic individual-based model, we here provide insights into how dispersal distance evolves in relation to the rate of climate change in an asexual plant species. We are interested in establishing whether the dispersal distance that evolves at an expanding front is the lowest that enables the population to track the changing climate. We also explore the degree to which these evolutionary changes allow populations to spread across gaps in the landscape and as such induce evolutionary rescue under the combined action of climate change and habitat fragmentation. While it can be expected that gaps are more readily crossed when climate change proceeds slowly because of an increased time window of opportunity and larger population sizes, we provide evidence of the opposite; somewhat counter intuitively, we show that slightly faster climate change can facilitate spread across fragmented landscapes because of evolution of increased dispersal distances. Furthermore, we emphasize that population spread projections developed from spatially stable landscapes, such as implemented in analytical wavespeed models (Neubert and Caswell 2000; Katul et al. 2005; Jongejans et al. 2008), may not be accurate predictions of range expansion ability under climate change.

## Methods

We developed a spatially explicit, individual-based model to investigate the evolution of dispersal kernels during range shifts. Simulations were run in discrete time and took place on a cellular lattice (*y* = 100, *x* = 1000) (see Figure S1 in Supporting Information for schematic representation). We used absorbing (i.e. lethal) boundaries because they are most appropriate for modelling passive dispersal (Burton and Travis 2008). We also tested a landscape without borders (torus), but patterns remained qualitatively similar (Figure S2 in Supporting Information).

### Population dynamics

We approximated the ecology of an annual plant species; within one generation adults produced a density-dependent number of seeds just before they die. These seeds inherit an allele from their parent which determines how wide their dispersal kernels are. Seeds will disperse a certain distance according to this kernel and survive to become adults if they settle in a suitable habitat that is exposed to the right environmental conditions (i.e. within the climate window). To keep things as simple as possible, we modelled reproduction as an asexual process. Within-population dynamics were based on well-understood density-dependent demographic processes (Hassell and Comins 1976). Each individual in a cell with local density *N* at time *t* gives birth to a number of offspring drawn at random from a Poisson distribution with mean *μ* calculated from the following expression:


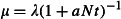


Here, *λ* specifies the net reproductive rate, *a* is a measure of patch quality and is defined as:


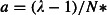


Where N* is the population equilibrium density; if the local density *Nt* is lower or higher than this value, the average number of offspring will increase or decrease, respectively, as a result of competition. The actual number of offspring *Λ* is drawn from a Poisson distribution with mean *μ*; as such demographic stochasticity is introduced into the model (Poethke and Hovestadt 2002; Travis and Dytham 2002; Travis et al. 2009). In our models, we used the parameter values *λ* = 2 and N* = 2, decreasing these values resulted in unviable populations, whereas increasing one of them improved population resilience. However, general patterns in our results remained unaltered (Boeye et al., unpubished data). We only allow plants to produce a few seeds, doing so we improve computational power and as such mimic low establishment success of seeds (Jakobsson and Eriksson 2000). There are no additional costs to dispersal in the base model except for the fact that the chance to end up outside the landscape or climate window inevitably increases with the dispersed distance, but we additionally modelled dispersal-dependent costs to constrain dispersal distances in a biologically meaningful sense (see sensitivity analysis). Survival and reproduction are only possible within suitable habitat inside the climate window. This window moves in the x direction at a speed varying from 0.05 to 6 grid cells/time step. By varying this rate, it is possible to simulate different rates of climate change. We used climate windows of 40 grid cells wide but also tested smaller (20 grid cells) and larger (80 grid cells) windows (see Figure S2).

### Evolution of dispersal

Each individual inherits a single allele from its parent which determines the shape of the individual's dispersal kernel defined as the parameter *δ*. More specifically the allele value (*δ*) determines the standard deviation of a Gaussian distribution with mean zero. Dispersal is then modelled by sampling displacement distances in two dimensions from this distribution (see Bonte et al. 2010). As the allele value describes a probability distribution rather than an exact value, the heritability of effective dispersal distance is <1, which is in line with empirical work (e.g. Bonte and Lens 2007; Cheptou et al. 2008; Bitume et al. 2011). We use *δ* as a measure for dispersiveness because individuals with high *δ* values have wide kernels with approximately 32% of the population moving beyond distance *δ* (principal characteristic of a Gaussian distribution). Individuals with a kernel with high *δ* consequently have a higher probability to disperse a long distance (see Table 1). For ease of reading, we will refer to this kernel parameter as dispersal distance. As we assume for simplicity uninformed, passive (wind) dispersal, long-distance dispersers from the tail of the kernel have a relatively high chance to disperse out of the population's suitable range, but this probability depends largely on the size of the climate window. When the model is initialized, each individual's allele value is set as a random value from the uniform distribution between 0 and 10. This leads to high standing genetic variation and allows spatial sorting to act. We also ran simulations after 500 generations of dynamics in a stable range, combined with changes in mutation rate thereby decreasing the level of standing genetic variation to derive the sensitivity of our conclusion regarding evolutionary rescue (see Table 2). Mutations on the allele occur with a probability of 1% in the base model and are randomly drawn from a uniform distribution (−1, 1). As a reference we determined invasion speed of populations with a fixed dispersal distance in landscapes without climate change, we kept the kernel parameter fixed and did not allow any mutations, thereby precluding evolution.

**Table 1:** Average and longest dispersal distance of 10 000 seeds with a certain ‘dispersal distance’ δ^*^ i.e. the standard deviation of a Gaussian kernel

δ^*^	Average distance	Longest distance
0.5	0.6	2.2
1	1.3	5.0
2	2.5	9.2
3	3.8	15.6
4	5.0	17.5
5	6.3	21.9
6	7.5	25.5
7	8.9	30.1
8	10.0	36.4
9	11.3	43.5
10	12.5	47.4

**Table 2:** The average success rates of 100 populations which had to track a moving climate window and cross a gap of unsuitable habitat in different scenarios. Note that in all (viable) scenarios, the success rate initially increases as the climate window moves faster (i.e. evolutionary rescue). Colour code: dark red = 0, bright red = 0.1–0.24, orange = 0.25–0.49, yellow = 0.5–0.74, bright green = 0.75–0.99, dark green = 1

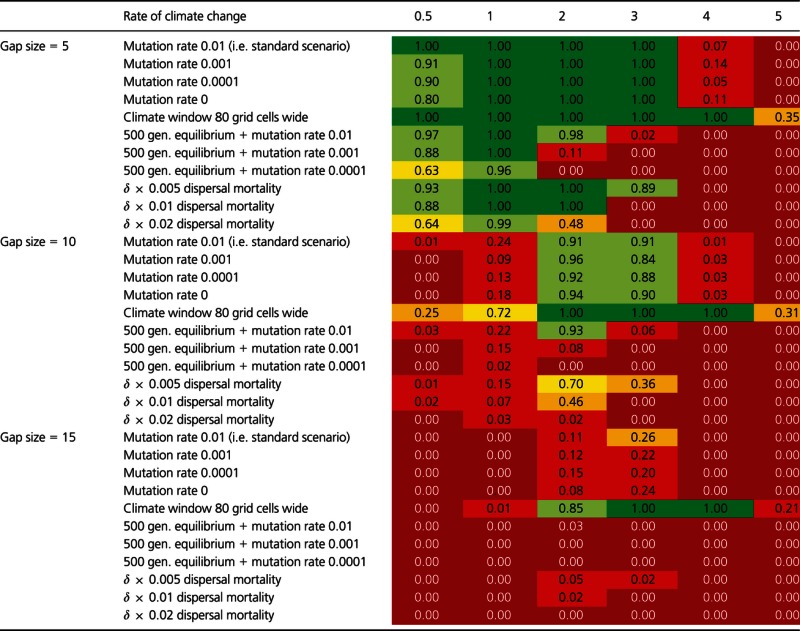

### Maximal tolerance of climate change and invasion speed

If we allow evolution of the dispersal distance, we expect that for each viable rate of climate change, an optimal, evolved, dispersal distance should arise over time. We compare the rate of climate change under which a certain dispersal distance allele (*δ*) has evolved to the fastest rate of climate change that population could track if the same dispersal distance allele (δ*) was fixed and equal in all its individuals. We call the latter rate the maximal ‘tolerance’ of climate change, and it is assessed as the maximal rate of climate change that a population with a genetically fixed dispersal distance allele (δ*) can keep up with over the whole length of the landscape during 30 runs without going extinct once. Next, we compare this rate to the speed at which the same population can invade empty habitat. It makes intuitively sense that a population which can invade empty habitat at a certain speed could shift its range equally fast when it is forced to by a climate window; therefore, both rates are expected to be similar. The invasion speed is defined as the average speed of the invasion front, calculated over 30 runs. Note that when we use a fixed dispersal distance parameter, it is not the dispersal distance itself that is fixed but the dispersal kernel shape (see earlier), we always denote fixed dispersal distance values with a ‘*’.

### The influence of the rate of climate change on gap-crossing capacity

To test the degree to which the speed of the moving envelope (rate of climate change) affects the probabilities that a shifting population crosses unsuitable habitat, we introduced a gap into our virtual landscape. Therefore, we considered an area of habitat from position *x* = 900 onwards as unsuitable habitat in the baseline model (see Figure S1). The width of this gap was fixed but varied between different scenarios (see Table 2). We ran the simulation 50 times for each combination of climate window speed and gap size. During these replications, we measured how often the population succeeds in crossing the gap.

To assess how population size changes and the dispersal distance (*δ*) evolves during such a simulation, we chose one specific set of parameter values and studied it in more detail. We moved the climate window at two grids cells/time step and used a gap width of seven grid cells. We repeated this simulation 100 times and calculated average population size and dispersal allele value (*δ*) for each time step. This simulation slightly differed from the base model as we did not move the climate window during the first 500 time steps, allowing us to check how this affects the results. After 980 time steps, the climate window reached the gap.

## Results

### The rate of climate change a species can track is lower than the rate at which it can invade

**Figure 1:**
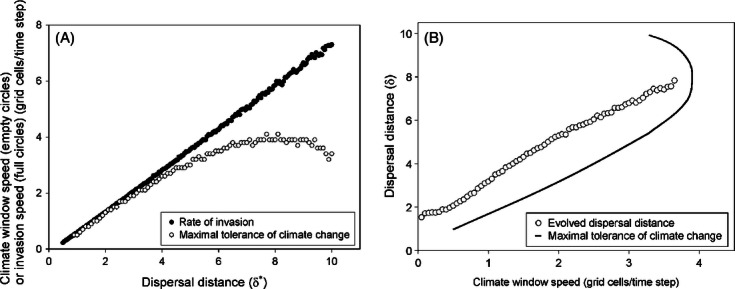
(A) The rate at which a population with a fixed dispersal distance parameter (δ*) can invade an empty spatially stable landscape (full circles) and the maximal climate window speed a population with the same dispersal kernel can track (empty circles). (B) Impact of climate window speed on evolved dispersal distance *δ* (open circles). The solid line depicts the maximal tolerance of climate change as depicted in Fig. 1A.

The rate at which a population can expand in a landscape without a climate window (invasion speed) is, as expected for a pulled front, linearly correlated to the implemented dispersal distance parameter δ*. At lower δ* values, there is also a linear relationship with the maximum climate window rate a population can tolerate without going extinct. However, at high dispersal distances (δ* > 6), this relationship does not hold; indeed higher δ* does not allow persistence in scenarios of faster climate changes and perhaps counter intuitively, the maximum rate of climate change that a species with very high δ* can tolerate may be lower than that which a species with lower δ* can tolerate (Fig. 1A). There is thus a divergence between invasion speed as determined in a spatiotemporally stable (empty) landscape and the maximal rate of climate change that a population with the same δ* can keep track with. The extent of this divergence grows with an increase in the dispersal distance parameter δ*.

### Evolved dispersal distance increases with the rate of climate change and is higher than necessary

**Figure 2:**
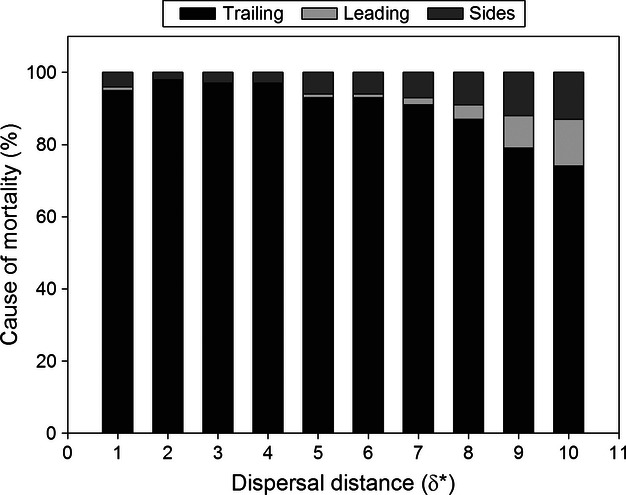
The proportional causes of mortality in a number of simulations with parameter values derived from the results in Fig. 1A. When dispersal distances are high, a relatively larger number of individuals land in front or to the sides of the climate window (as a seed) and die.

**Figure 3:**
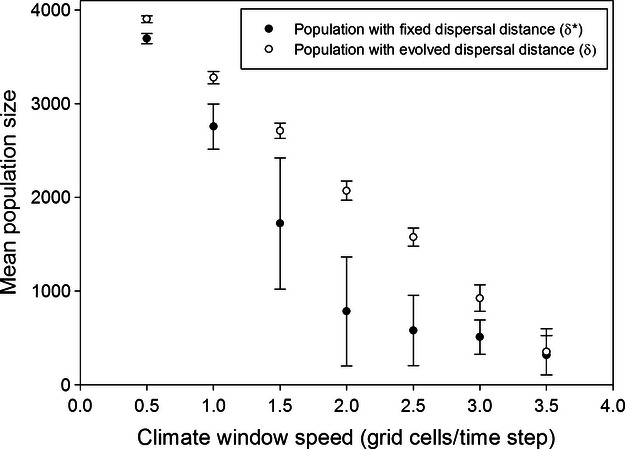
The difference in average population size between populations having the lowest fixed dispersal distance parameter (δ*) that allows tracking the climate window and evolved dispersal distance (*δ*) for several climate window speeds. The error bars denote the standard deviation based on 10 replicas.

Increasing rates of climate change induce evolution towards higher dispersal distances *δ* (Fig. 1B). Comparison of the average evolved dispersal distance *δ* with the lowest dispersal distance δ* that allows a population to track the shifting window without going extinct (full line from Fig. 1B), indicates that evolved dispersal distances are always higher than is absolutely necessary for tracking a shifting climate window. For each rate of climate change, there is selection for the genotypes that optimally balances dispersal mortality and the capacity to track the climate window, resulting in a maximization of the population size (Figure S3). When the speed of climate change exceeds 3.7 grid cells/time step, the combination of high mortality by the trailing edge of the climate window and high mortality of long-distance dispersers pushes the population to the limit of what is theoretically possible in our model. This is why there is no crossing of the full line with the open symbols in Fig. 1B. Evolution can thus only allow individuals to keep track of climate change until a critical climate change rate. Under higher rates of climate change, dispersal distance *δ* evolves to such values that mortality because of ending up outside the climate window, at the leading, trailing edge, but also at side edges becomes too high (Fig. 2). Increased costs of dispersal, here implemented by inducing higher rates of mortality because of ending up outside the suitable range, thus constrain the capacities to keep track of a shifting climate envelope. When the climate window moves slowly, short dispersal distances evolve and the trailing edge accounts for almost 100% of deaths, while at higher rates of climate change, and the subsequent evolved high dispersal distances, mortality due to crossing the leading or side edges becomes more substantial (Fig. 2). We present an animation of the spatial distribution of individuals within the climate window for different rates of climate change and species’ dispersal distance (see Figure S4). These dynamics are also influenced by the size of the landscape, with reduced costs of ending up aside the landscape in wider or in continuous landscapes modelled as a torus. This implies that the evolution towards higher dispersal distances will be easier in populations that occupy a large distribution range or face lower dispersal costs, thereby allowing individuals to keep track of faster moving climate windows (Figure S2). Populations that have smaller ranges due to, for instance, local adaptation towards specific climatic conditions will be subject to an even stronger selection for higher dispersal distances but are less feasible to persist because they are more likely to disperse into an unsuitable environment. Simulations with evolved dispersal distances always resulted in larger population sizes than equivalent simulations where instead we used the lowest fixed dispersal distance parameter δ* that allowed tracking of the climate window (Fig. 3).

### High variability in dispersiveness is maintained in a moving climate window

**Figure 4:**
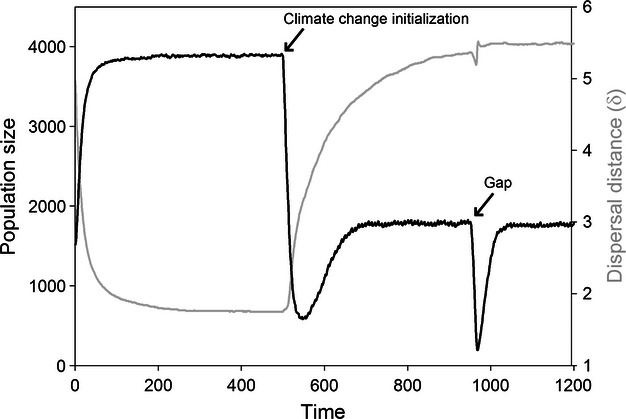
The average population size (black line) and average dispersal distance (*δ*) (grey line) over time. During the initial 500 time steps without climate change, the average dispersal distance decreases and reaches equilibrium. As soon as the climate window starts to shift, the dispersal distance increases rapidly. There is a small drop in average dispersal distance when the climate window is reached. The population size crashes initially but eventually recovers and stabilizes at less than half the population's size without climate change. When a gap in the landscape is reached, the population almost goes extinct but eventually recovers.

**Figure 5:**
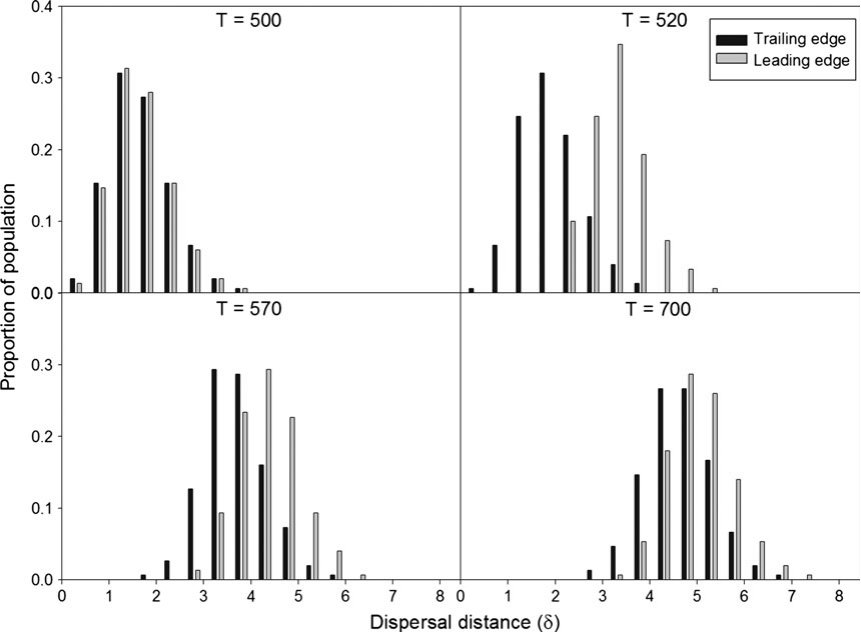
The average frequency of dispersal distance genotypes near (distance < 5 grid cells) the leading-(grey) and trailing edge (black) of the climate window at four different moments in time (*T*) calculated over 1000 simulation runs. In this model, the climate window only started moving after 500 generations (*T* = 500), the upper left figure thus gives us the equilibrium distribution of genotypes before climate change. There is a strong selection favouring more dispersive genotypes when the climate window starts to shift (*T* = 520, 570). Which eventually results in a stable frequency-distribution of genotypes after 200 generations of climate change (*T* = 700). For this specific model, we used a climate window moving at two grid cells/time step.

After 500 time steps (generations) without climate change, average dispersal distance allele values are strongly reduced (see Fig. 4); however, kin competition withholds the dispersal distance from evolving to zero. At this stage, only a few distant dispersal genotypes (*δ* > 3) remain (Fig. 5). After the onset of climate change, these genotypes become more abundant relative to those that are less dispersive and new, even more dispersive, mutants arise. This pattern holds when decreasing mutation rates up to 10e−6. However, the maximal rate of climate change a population can track increases with the mutation rate (see Table 2). Soon after the initialization of climate change, a large difference in average dispersal distance allele values between leading- and trailing edge subpopulations arises, and this difference gradually diminishes over time but continues to exist. In both the subpopulations near the leading- and trailing edge, average dispersal distance allele values reach equilibrium after 200 time steps of climate change (*t* = 700). Even after the distribution of genotypes has stabilized, a remarkably large standing genetic variation in dispersal distance alleles remains, ranging from the least dispersive genotype that can tolerate a climate window moving at two grid cells/time step (*δ* = ±2.7 see Fig. 1A and Fig. 5 at *t* = 700) to much more dispersive genotypes.

### Faster climate change increases gap-crossing capacities of a population

**Figure 6:**
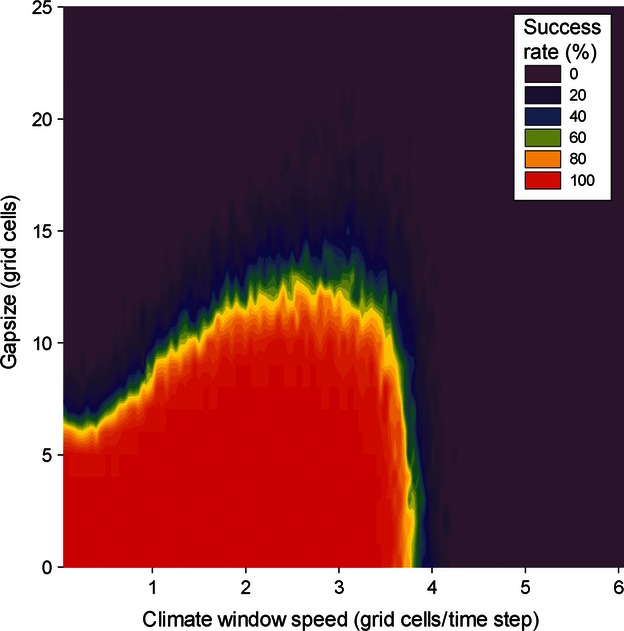
Success rate of gap crossing in populations with evolving dispersal distance (*δ*) according to the speed of climate change (*x*-axis) and gap size (*y*-axis). Faster moving climate windows induced selection for more dispersive genotypes and increased the probability of the population to cross the gap.

The speed of the shifting climate window has a pronounced impact on the gap size that can be crossed (Fig. 6). In absence of any climate change or at lowest climate change speed, the gap size that can be successfully crossed is around six units, gaps of twice that size can be successfully crossed at a climate window speed between 2 and 3.7 grid cells/time steps. At high climate window speeds (>3.7 grid cells per time step), the success rate drops drastically and eventually populations become extinct before they reach the gap. The exact outcome of this model was sensitive to a number of parameters and conditions but the qualitative pattern of temporarily increased persistence always remained prevalent (see Table 2).

## Discussion

By means of generic modelling, we show that (i) increased rates of climate change select for larger dispersal distances; (ii) evolved dispersal distances are higher than strictly necessary to keep track of the climate window and maximize population size; (iii) the maximal rate of climate change that a population can successfully track is lower than the rate at which a population expands in empty landscapes, not affected by a shifting climate window (invasion speed); (iv) the evolution of dispersal distance induces a rescue mechanism when gaps of unsuitable habitat need to be crossed during range expansion under climate change.

Dispersal kernels evolve towards larger displacement distances by both natural selection and spatial sorting when the rate of climate change increases. In accordance with previous studies on emigration rate (Travis and Dytham 2002; Travis et al. 2009; Burton et al. 2010), spatial sorting processes are most important at the onset of climate change, while natural selection on dispersal distance becomes the main mechanism at the leading edge. Interestingly, evolved dispersal distances are always higher than necessary for range expansion through invasion in a landscape without shifting climate windows. Populations characterized by a specific kernel will subsequently show larger range expansion in unoccupied landscapes when climate windows do not limit them. Classical invasions (Shigesada and Kawasaki 1997) do not impose the same limitations on population expansion as a climate window, that is, increased mortality because of overshooting the climate window dimensions and to a lesser extent mortality at the trailing edge (Cousens et al. 2008). Modelled invasion rates (Neubert and Caswell 2000; Katul et al. 2005) should therefore be applied with some caution to estimate the maximal rate of climate change a species can tolerate. Methods developed to predict the rate of expansion in empty habitat do not account for limitations in spatial dynamics under climate change and could thus overestimate the rate of climate change a species can track. In our model, the only difference between an invasion and a range shift with a moving climate window is the presence of two extra boundaries in a shifting climate window, thereby limiting the population's spread. In accordance with Pease et al. (1989), we showed that a larger distance between the leading and the trailing edge of the climate window allowed the populations to keep track of a faster moving climate window. A larger climate window decreased dispersal mortality and thus allowed the evolution towards higher dispersal distances. The opposite was true for a smaller climate window. In reality, this effect is likely to be experienced by populations that have narrow distribution ranges because of local adaptations to climate heterogeneity or the preference of a rare type of habitat. In these populations, highly dispersive individuals would have low survival chances because they incur a high risk of ending up in unsuitable habitat, at least in the case of passive dispersal. We implemented absorbing border conditions on the nonshifting edges of the climate window. Such absorbing boundaries strengthen the selection against long-distance dispersal (Burton and Travis 2008). Assuming no edge effects by wrapping boundaries using a torus did not, however, change the results in a qualitative way given the proportional marginal mortality effects at these edges relative to mortality at the trailing or leading edge.

Because of spatial sorting, even despite the absence of assortative mating and subsequent natural selection, a large difference in average dispersal distance (*δ*) between individuals near the trailing and leading edges occurs after 10–20 generations. From this point onwards, natural selection slowly starts excluding low fitness genotypes that are either not dispersive enough to consistently keep up with the window and highly dispersive genotypes that are too likely to disperse outside the window. This leads to a decreasing difference in dispersal distance between individuals from the trailing and leading edge, thereby generating stabilizing selection towards an optimal dispersal strategy and a maximization of the total population size (Figure S3). Spatial gradients in selection pressures inside the climate window generate a large standing genetic variation during range expansion, ranging from the least dispersive individuals that could track the window to much more dispersive individuals. This explains why the average dispersal distance allele value was higher than necessary to keep track of a certain rate of climate change. Near the leading edge, dispersive individuals with wide kernels have an advantage since they are more likely to colonize the empty habitat that constantly becomes available at this location (Travis et al. 2010; Phillips 2012). However, when approaching the trailing edge, population densities gradually grow and increase competition, thereby benefiting lineages consisting of shorter dispersal distance genotypes. Because wide dispersal kernels incur a cost of ending up beyond the window (Fig. 2), the eventual evolutionary stable dispersal distance (*δ*) will depend on the dimensions of the landscape. From earlier work, it is known that mortality because of low colonization success in unsuitable habitat at the edge of a population's distribution is a mechanism of range border formation (Holt and Keitt 2000). According to the landscapes dimensions, a threshold point of climate change speed has been observed beyond which populations become too small to remain viable during the process of tracking the climate window.

Evolutionary rescue is the process where the increase in a few well-adapted genotypes can counter the decline of a maladapted population during a period of environmental change (see Ferrière et al. (2004) for theory), and typically results in a U-shaped function of population size over time (Holt and Gomulkiewicz 2004). The potential importance of this process in conservation biology has been topic of several theoretical (Heino and Hanski 2001; Travis et al. 2010) and empirical studies (Bell and Gonzalez 2011). In our study, somewhat higher rates of climate change increase the capacity of a population to cross gaps in the landscape during climate-driven range expansion for a wide range of parameter space (Table 2). As such, slightly faster climate change may induce evolutionary rescue (Clobert et al. 2001) for species experiencing locally fragmented habitat (Fischer and Lindenmayer 2007).

A first evolutionary rescue event takes place at the onset of climate change. Under these conditions, only dispersive genotypes survive (and thrive) and low population sizes are overcome (Fig. 4). The second rescue event, gap crossing, is enhanced at higher rates of climate change and again a typical U-shape in population size is observed with only highly dispersive individuals making it across the gap (Fig. 4). Population history subsequently strongly affects this second rescue event (Phillips 2012). Of course, these rescue mechanisms will only be relevant in species and/or populations showing sufficient standing variation in dispersal traits (Pease et al. 1989) through, for instance, diverging selection pressures in heterogeneous landscapes (Bonte et al. 2010). However, while not a focus of this study, local adaptations in heterogeneous landscapes could in turn impede range shifts trough migration load (Polechová et al. 2009; Atkins and Travis 2010; Duputié et al. 2012). In theory, we might make the initially counterintuitive suggestion that those species that have long life cycles may benefit most from the dispersal enhancing selection pressure that facilitates gap crossing as they experience time and thus the rate of climate change faster (the generation effect). Similar rescue mechanisms may be equally more relevant for species living in biomes characterized by fast climate change like savannah compared to biomes that are subjected to relative slow climate change like tropical coniferous forest (Loarie et al. 2009), at least if range expansion and evolution do occur in more continuous suitable landscape.

Traits determining dispersal distance are shown to have a genetic basis and subject to multiple costs (Bonte et al. 2012). While the evolvable maximal dispersal distance is expected to be constrained because of morphological, physiological and life-history trade-offs (Travis et al. 2012), our simulations demonstrate that the evolution towards increased dispersal distances may rescue species up to specific limits that are determined by dispersal costs, the level of standing genetic variation and the landscape context (here size of the gap and climate window). The loss of genetic variation during a phase of genetic equilibrium without a shifting climate window additionally decreases evolutionary rescue probabilities and increases the sensitivity towards low mutation rates (Table 2). The exact rates of climate change which could induce evolutionary rescue are therefore likely to differ strongly among species. So, while there is currently a consensus that too fast climate change will be detrimental for many species (e.g. Visser 2008; Berg et al. 2010), our modelling approach shows that under an increased rate of climate change that does not generate direct extinction, evolutionary dynamics in dispersal are likely to induce rescue mechanisms especially in landscapes that suffer from rather limited habitat fragmentation. While it will be extremely challenging to predict which species may be rescued by evolutionary dynamics, our results at least should make it possible to identify species that will face problems in keeping track with increasing rates of climate change, that is, species experiencing distance-related dispersal costs, having small distribution ranges, limited genetic variation in traits determining dispersal distance and/or experiencing large barriers in the landscape or too high rates of climate change relative to their dispersal distance.

Populations facing climate change need to adapt to the new environment or track the climate window to avoid extinction (Visser 2008). Here, we demonstrate the importance of combined responses, changes in the dispersal kernel as an adaptation. We show that fast climate change can induce selection for wider dispersal kernels, as such ensuring population persistence and even evolutionary rescue in case of range shifting in fragmented areas. Interestingly, our model demonstrated a discrepancy between the rate of climate change a population can tolerate and the rate at which the same population can invade empty habitat. This warns us to be careful when estimating the maximal rate of climate change a species can tolerate based on the invasion speed of that species. While the impact of climate change rate on range expansion and dispersal evolution is clear from a theoretical point of view, processes are expected to be much more complicated in reality because of trade-offs in life-history traits (Burton et al. 2010), multiple species interactions (Urban et al. 2012) and several (novel) costs involved during the dispersal process (Bonte et al. 2012; Travis et al. 2012). Model approaches like applied here are, however, a first and important step to understand the huge variation in range shifting patterns relative to life-history traits like dispersiveness, reproductive ability and ecological generality (Angert et al. 2011).

## Supporting Information

**Figure S1.** Schematic representation of the modelled landscape.

**Figure S2.** Sensitivity analyses according to landscape and climate window properties.

**Figure S3.** Comparison of population sizes between populations with a fixed or evolving dispersal distance d at different rates of climate change.

**Figure S4.** Animation of the spatial distribution of individuals within the climate window for different rates of climate change (CWS) and different fixed values of dispersiveness (*δ**).
