# Supplementary material for: More rapid climate change promotes evolutionary rescue through selection for increased dispersal distance
Source: Evol Appl. 2012 Sep 25;6(2):353–64. doi: 10.1111/eva.12004 (PMC3586623; doi:10.1111/eva.12004)
Supplement: Supplementary file 1 [file eva0006-0353-SD1.pdf]

Figure S1 Schematic representation of the modelled landscape.

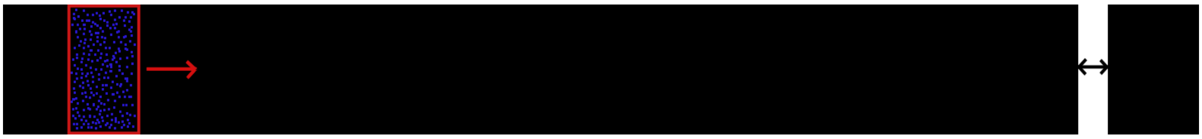

Individuals (blue dots) can survive and reproduce in a patch that both consists of suitable habitat (black) and is located in the moving climate window (red rectangle). In some cases the population is confronted with a gap of unsuitable habitat (white) at the end of the landscape. The width of this gap is fixed during a single simulation but is systematically varied over several runs.
