# Supplementary material for: More rapid climate change promotes evolutionary rescue through selection for increased dispersal distance
Source: Evol Appl. 2012 Sep 25;6(2):353–64. doi: 10.1111/eva.12004 (PMC3586623; doi:10.1111/eva.12004)
Supplement: Supplementary file 3 [file eva0006-0353-SD2.pdf]

Figure S2 Sensitivity analyses according to landscape and climate window properties.

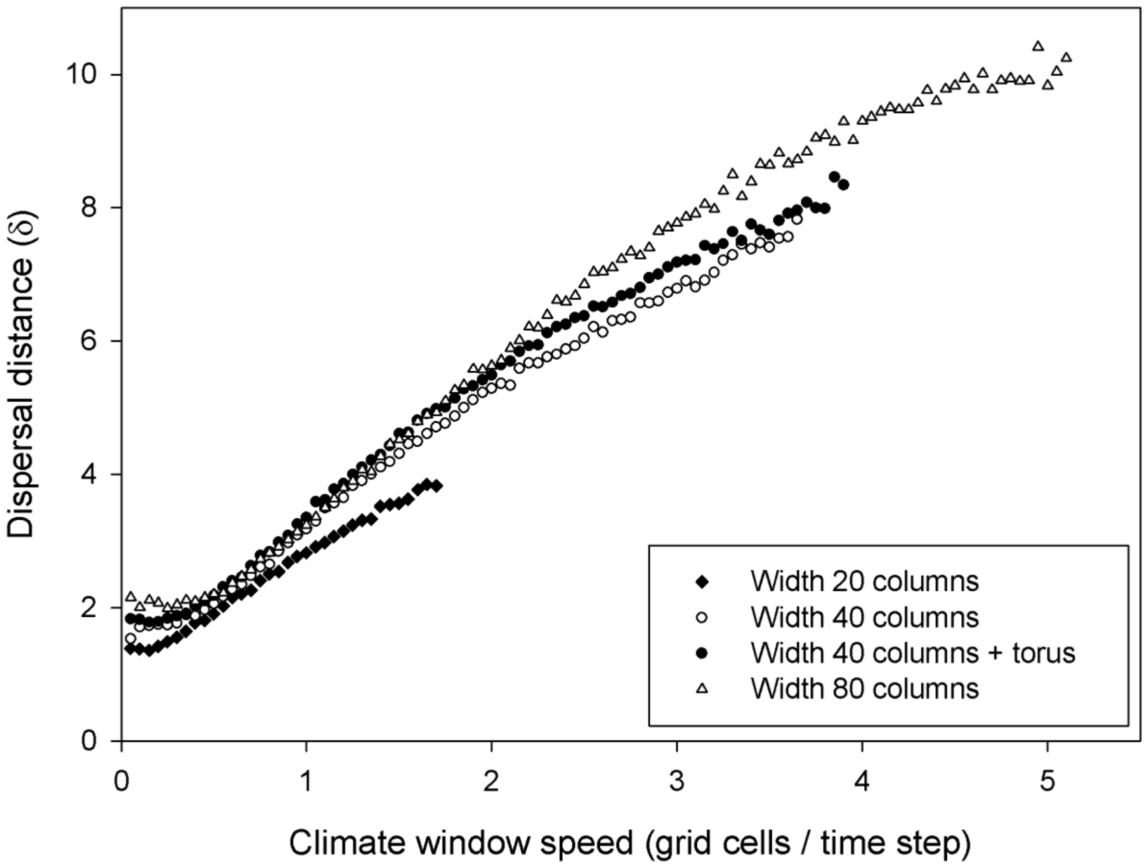

The evolved dispersal distance as a response to different sizes and velocities of the climate window. The distance between the leading and trailing edge of the window was varied. Larger climate windows allow populations to persist in environments that change faster. Usually climate windows are 40 grid cells wide in the x-direction (direction of movement) and 100 grid cells long in the y-direction, if we make the length of the climate window infinite in the y-direction (i.e. a torus) there is only a small positive effect on evolved dispersal distances and maximal rate of climate change the population can track.
