# Supplementary material for: More rapid climate change promotes evolutionary rescue through selection for increased dispersal distance
Source: Evol Appl. 2012 Sep 25;6(2):353–64. doi: 10.1111/eva.12004 (PMC3586623; doi:10.1111/eva.12004)
Supplement: Supplementary file 5 [file eva0006-0353-SD3.pdf]

Figure S3 Mean population size over several rates of climate change for fixed and evolving *δ*.

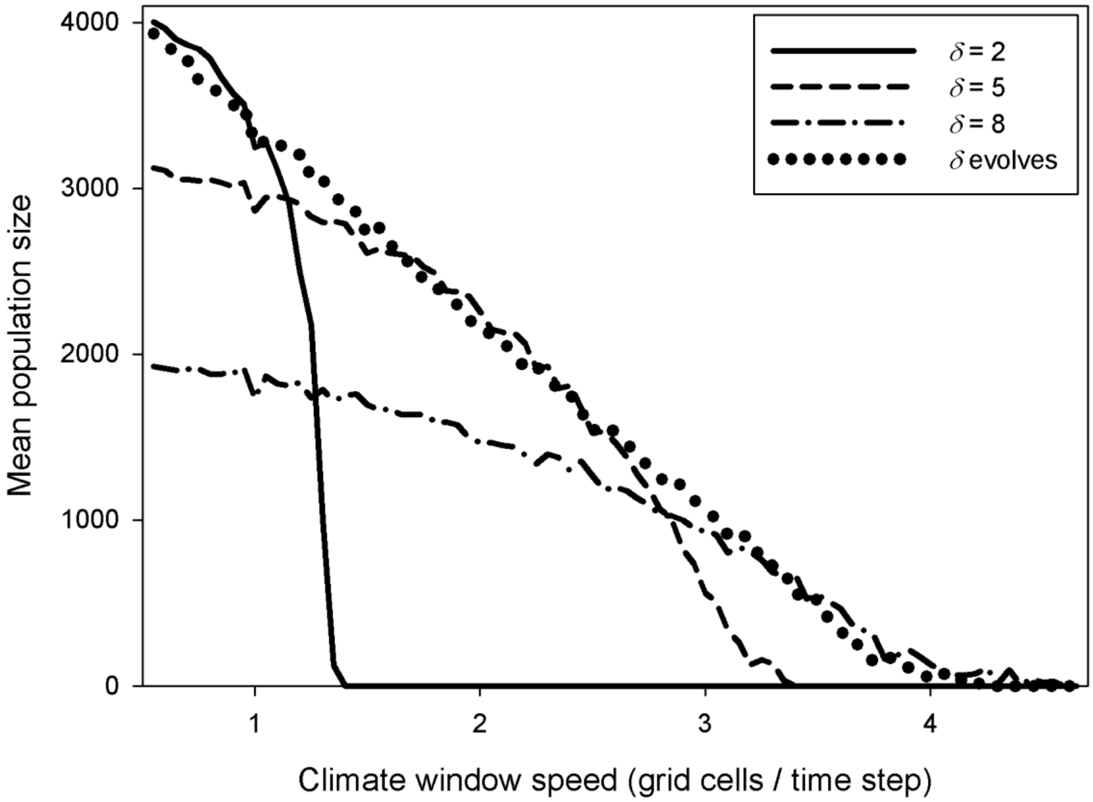

When dispersal distances (*δ*) are fixed there is a clear trade-off between population size at low rates of climate change and the capacity to cope with high rates of climate change. This is due to higher mortality of dispersive individuals in slow moving climate windows and high mortality of poor dispersers in faster moving climate windows. When the dispersal distance (*δ*) is allowed to evolve there is a selection for the dispersal distance that finds an optimal balance between dispersal mortality and resilience to the shifting climate window. This results in a maximization of the population size for each rate of climate change.
