# Supplementary figures and images for: More rapid climate change promotes evolutionary rescue through selection for increased dispersal distance
Source: Evol Appl. 2012 Sep 25;6(2):353–64. doi: 10.1111/eva.12004 (PMC3586623; doi:10.1111/eva.12004)

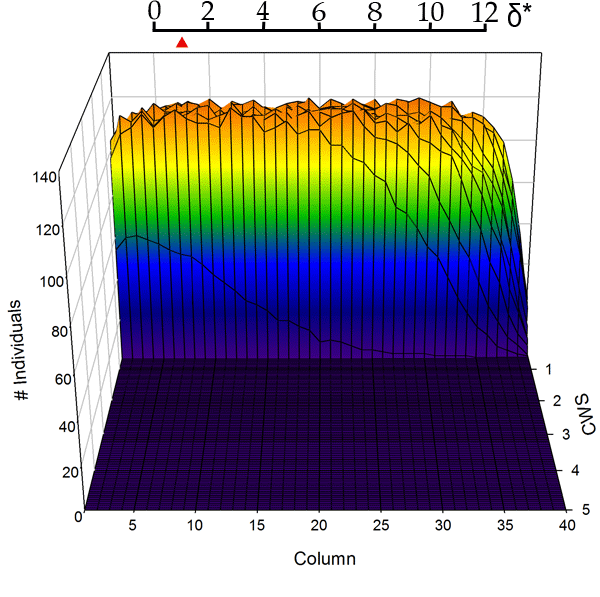

Supplement: Supplementary file 7 [file eva0006-0353-SD4.gif]
